# Correlation between PAPP-A serum levels in the first trimester of pregnancy with the occurrence of gestational diabetes, a multicenter cohort study

**DOI:** 10.1186/s12884-023-06155-7

**Published:** 2023-12-11

**Authors:** Sedigheh Borna, Masoumeh Ashrafzadeh, Marjan Ghaemi, Nasim Eshraghi, Nafiseh Hivechi, Sedigheh Hantoushzadeh

**Affiliations:** Vali-E-Asr Reproductive Health Research Center, Family Health Research Institute, Tehran University of Medical Sciences, Tehran, Iran

**Keywords:** Pregnancy associated plasma protein A, Gestational diabetes, Pregnancy

## Abstract

**Objective:**

This study aimed to investigate the association between first-trimester Pregnancy-associated plasma protein A (PAPP-A) levels and subsequent gestational diabetes mellitus (GDM) development.

**Method:**

The study was conducted on 5854 pregnant women who attended routine prenatal care. Maternal biomarkers, including PAPP-A and free beta hCG, were measured for all women in a referral laboratory and converted to MoM values. Pregnant women were divided into two groups, based on the serum concentration of PAPP-A, (PAPP-A > 0.4 (normal) and PAPP-A < 0.4 (low)). Data on the screening test for GDM and pregnancy outcomes were collected and analyzed with appropriate tests.

**Result:**

Of the 5854 pregnant women, 889 (15.19%) developed GDM. The maternal PAPP-A MoM concentrations were significantly lower in GDM cases compared to controls. Indeed, gestational age at delivery and birth weight were significantly lower (p < 0.001) in PAPP-A MoM < 0.4, and the rate of intrauterine growth restriction (IUGR) was significantly higher (p < 0.001). ROC analysis revealed that the sensitivity and specificity of MoM concentration for predicting GDM were 53.3% and 51.9%, respectively.

**Conclusion:**

Lower maternal PAPP-A in early pregnancy can lead to glucose intolerance and increase the risk of subsequent GDM development. In addition, decreased serum concentration of PAPP-A is significantly correlated to lower birth weight and IUGR.

## Introduction

Gestational diabetes mellitus (GDM) is defined as a glucose intolerance detected for the first time in the second or third trimester of pregnancy in the absence of overt diabetes [1, 2]. GDM can negatively affect pregnancy and lead to complications such as preeclampsia, polyhydramnios, fetal macrosomia, shoulder dystocia, and cesarean Sect. [3]. All pregnant women should be tested for gestational diabetes at 24–28 weeks of gestation with a 75-g OGTT [4].

GDM pregnant women may have high blood glucose levels before the diagnosis at 24 weeks of gestation, therefore fetal growth could be adversely affected by maternal hyperglycemia [5] using first-trimester screening maternal serum biomarkers may lead to early diagnosis and interventions for GDM to improve maternal and fetal health outcomes [6].

GDM cases had lower serum pregnancy-associated plasma protein (PAPP-A) [7]. PAPP-A is a high molecular weight protein secreted in high concentration from the placental syncytiotrophoblast and is routinely measured during the first trimester to determine the risk of aneuploidy [8]. PAPP-A levels are known to be associated with placental size or defective syncytiotrophoblast development [9, 10]. Also, it is indicated that the occurrence of several adverse pregnancy outcomes, including fetal loss, preterm birth, gestational hypertension, preeclampsia, and low birth weight, are more prevalent when the first-trimester PAPP-A levels are lower [11].

PAPP-A is a cleaving enzyme for IGF-binding proteins (IGFBPs) and regulates IGF-I bioavailability [12] which is a crucial factor for the control of maternal glucose hemostasis. A decline in first-trimester maternal PAPP-A could be related to glucose intolerance subsequently [3]. Screening of PAPP-A in pregnant women may be a predictor for GDM.

Because of the high prevalence of GDM among Iranian women [13] as well as the importance of early diagnosis, it is beneficial to identify a proper cost-effective screening test for early prediction of GDM during pregnancy. Some recent studies have indicated that low serum concentration of PAPP-A is related to the later development of GDM in the second and third trimesters. Therefore, the measurement of PAPP-A could help to identify the women who have an increased risk of GDM [7, 14, 15]. In this study, our objective is to evaluate the relationship between PAPP-A levels during the first trimester and the development of GDM in subsequent months.

## Materials and methods

### Study setting

This prospective cohort study enrolled pregnant women who visited perinatology clinics at four academic centers for first-trimester screening tests between March 2020 and March 2022. The referral laboratory assessed biochemistry markers, including PAPP-A and free beta hCG, and the timing of PAPP-A measurement was consistent for all participants (between 11 and 14 weeks of gestation). The inclusion criteria were singleton pregnant women aged 18–45 years’ old who underwent first-trimester pregnancy evaluation for aneuploidy. exclusion criteria included multiple gestations, fetal anomalies, pre-gestational diabetes, pre-gestational hypertension, nephropathy, impaired thyroid function, use of corticosteroids or immunosuppressive medications, and hormonal therapy.

### Ethical consideration

The institutional review board of Tehran university of medical sciences (TUMS) approved this study, which adhered to the Hesinki declarations. All participants provided informed consent. (No: IR.TUMS.ILHC.1400.526)

### Participant recruitment

Patient characteristics such as age, pre-gestation body mass index, parity, smoking status, and mode of conception, were recorded. Additionally, data on serum concentration of PAPP-A and free β-hCG were collected. Gestational age was calculated using crown rump length ultrasound measurement (CRL) or the last menstrual period. pregnancy outcomes at delivery were also documented. Multiples of the median (MoM) of PAPP-A and free β‐hCG were adjusted by maternal characteristics, including weight, height ethnicity, and smoking [16]. participants were categorized into two groups based on their PAPP-A levels: (1) those with MOM PAPP-A > 0.4 (normal) and (2) those with MOM PAPP-A < 0.4 (low) [17].

The cases received the same antenatal care and were monitored throughout their pregnancies. All participants underwent a screening test for gestational diabetes mellitus with 75-g glucose between 24 and 28 weeks of gestation The diagnosis of GDM was confirmed if the glucose level equaled or exceeded 92 mg/dl, 180 mg/dl, 153 mg/dl for the fasting, 1 and 2 postprandial respectively [18]. Pregnant women with GDM were treated with lifestyle modification or medical therapy.

### Statistical analysis

The participants’ information was collected securely and only the necessary data was imported to SPSS. Missing data was not replaced. Analysis of data was performed by using SPSS (version 26.0, SPSS Inc., Chicago, IL, USA). Qualitative variables were presented as frequency distribution while Normal quantitative data were presented by mean (standard deviation (SD)) and median (interquartile range (IQR)) was used to present non-normal data. The two groups were compared using an independent t-test or Mann-Whitney U test. The chi-square test or Fisher’s exact test was used to compare the categorical variables. A significance level of p < 0.05 was used. Receiver operating characteristic curve analysis was performed to determine the sensitivity and specificity of PAPP-A in predicting GDM.

## Result

A total of 6457 pregnant women with a singleton pregnancy were included in this study (603 pregnant women were excluded because of existing missing data). Of these, 889 (15.19%) cases had GDM and 4965 (84.81%) were non-GDM. The participants were divided into two groups, based on the PAPP-a level and maternal characteristics, and pregnancy outcomes were compared between 322 pregnant women with low PAPP-a level and 5532 participants with normal PAPP-a. The results were demonstrated in Table 1. Maternal age (p = 0.035), weight (p = 0.024), and BMI (pre-gestational) (p < 0.001) were significantly higher in the low PAPP-A group respectively.

There was no statistically significant difference between the two groups in the number of weight gains during pregnancy (p = 0.118), the rate of IVF (p = 0.977), and smoking (p = 0.760). The number of null gravidae was significantly higher among women with increased PAPP-A (p = 0.008). At delivery, the gestational age was significantly lower in the low PAPP-A group (p < 0.001). Notable differences were detected when the two groups were compared regarding birth weight (p < 0.001) and the rate of IUGR (p < 0.001).

**Table 1:** Maternal and pregnancy characteristics of the study population

Characteristics		PAPP-A MoM < 0.4 (n = 322)	PAPP-A MoM ≥ 0.4 (n = 5532)	p-value
Maternal age (year)		32.51 (4.83)	31.91 (± 5.02)	0.035
Weight (kg)		70.18 (± 10.88)	68.68 (± 11.54)	0.024
BMI (Kg/m2)		26.53 (± 3.86)	25.67 (± 4.09)	< 0.001
Weight gain during pregnancy (Kg)		13.28 (± 5.65)	13.83 (± 5.79)	0.118
Smoker		3 (0.93%)	43 (0.78%)	0.760
IVF		32 (9.94%)	547 (9.89%)	0.977
Nulligravida		135 (41.92%)	2741 (49.54%)	0.008
Fetal sex	female (n = 2740)	122 (4.44%)	2618 (95.55%)	0.503
	male (n = 3114)	158 (5.07%)	2956 (94.93%)	
Gestational age at delivery (week)		36.42(± 5.69)	37.65 (± 3.51)	< 0.001
Birth weight (gr)		3007.26 (± 523.07)	3135.49 (± 488.98)	< 0.001
IUGR		45 (13.97%)	485 (8.77%)	< 0.001

Data are presented as Mean (± SD, range) or n (%) or Median (range). The t-test was employed to compare the numerical variables between groups, while the Pearson chi-square test was utilized to compare the categorical variables among the groups

Body mass index (BMI), In vitro fertilization (IVF), Intrauterine growth restriction (IUGR), Pregnancy-associated plasma protein (PPAP-A), Multiple of the median (MoM)

The median (Interquartile range (IQR)) of PAPP-A MOM in the GDM women was 0.93 (0.59–1.38) and among non-GDM participants was 1.00 (0.67–1.49), also low serum PAPP-A MOM level was associated with GDM (p = 0.043). But the free-βhCG level and nuchal translucency length were not significantly different between the GDM and non-GDM women (p = 0.905, P = 0.313, respectively) (Table 2).

**Table 2:** Median values of maternal serum biomarkers and nuchal translucency length in women with GDM and normal pregnancies

	GDM group (n = 889)	non-GDM group (n = 4965)	P- value
free-βhCG MoM, median (IQR)	1.10 (0.70–1.71)	1.08 (0.73–1.68)	0.905
PAPP-A MoM, median (IQR)	0.93 (0.59–1.38)	1.00 (0.67–1.49)	0.043
Nuchal translucency (mm), median (IQR)	1.60 (1.37–1.80)	1.60 (1.30–1.80)	0.313

Data are presented as median (IQR). The mann-Whitney test was employed to compare the numerical variables between groups

Gestational diabetes mellitus (GDM), free β-human chorionic gonadotropin (fβ-hCG), Interquartile range (IQR), Multiple of the median (MoM), Pregnancy-associated plasma protein A (PPAP-A)

A binary logistic regression analysis was conducted to assess the risk factors for GDM, and the results are presented in Table 3. The analysis indicated that several independent risk factors were significantly associated with GDM, including lower PAPP-A MoM (OR = 0.890, P = 0.020), older maternal age (OR = 1.050, P < 0.001), greater maternal weight (OR = 1.029, P < 0.001), and a higher number of gravidity (OR = 1.079, P = 0.035). Although the risk of developing GDM was higher among pregnancies resulting from in vitro fertilization, this association did not reach statistical significance (P > 0.05).

**Table 3:** Investigation of the risk factors for GDM using binary logistic regression analysis

	OR	95%CI	SE	P value
Maternal age	1.050	1.034–1.067	0.008	< 0.001
Maternal weight	1.029	1.023–1.035	0.003	< 0.001
Gravidity	1.079	1.006–1.158	0.036	0.035
IVF	1.245	0.984–1.574	0.120	0.068
PAPP-A MoM	0.890	0.808–0.982	0.050	0.020

Binary logistic analysis was used to evaluate the relationship between GDM and maternal factors

Odds ratio (OR), Confidence interval (CI), Standard error (SE), In vitro fertilization (IVF), Multiple of the median (MoM), Pregnancy-associated plasma protein A (PPAP-A)

**Fig. 1:**
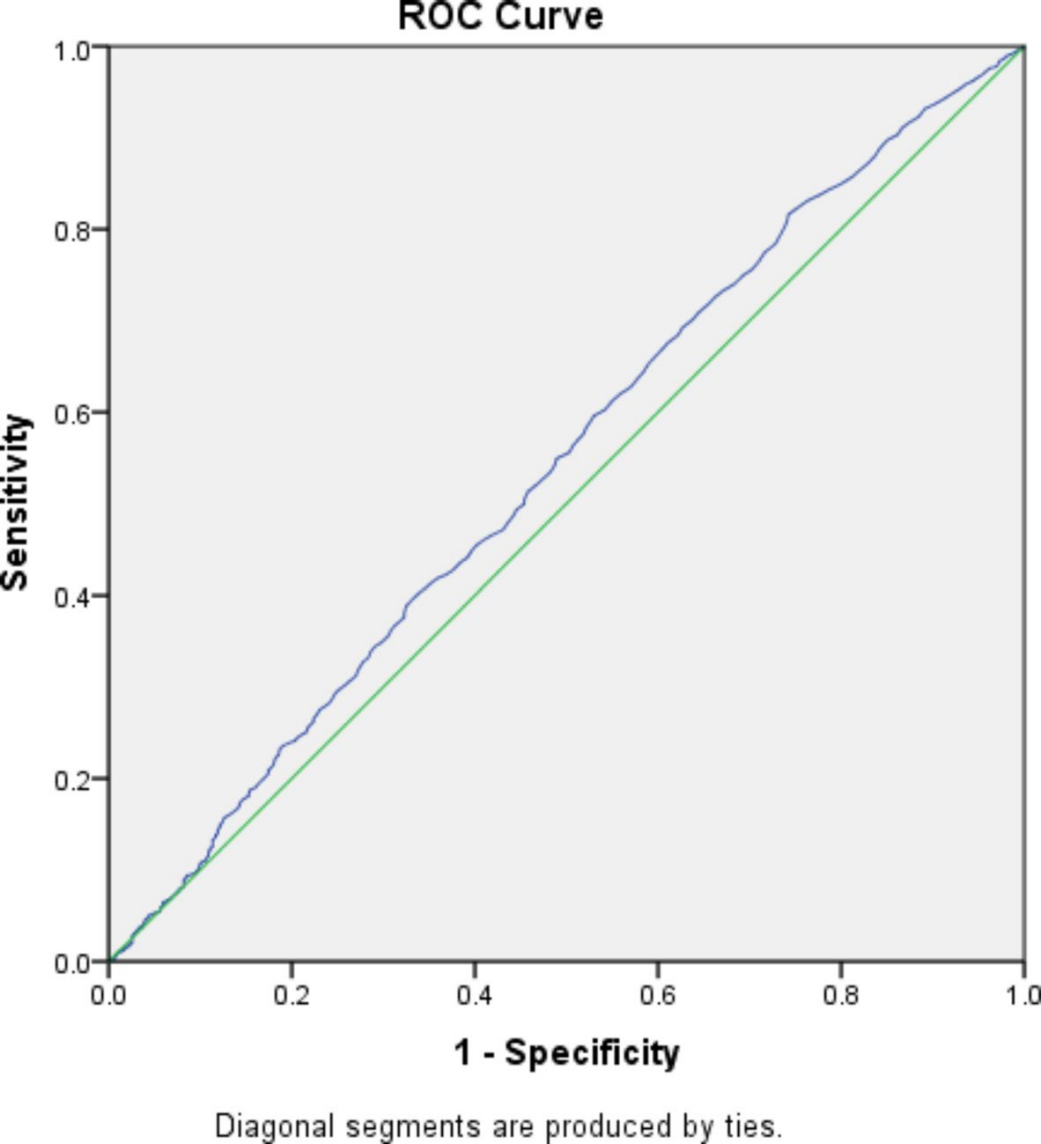
Roc curve statistics for PAP-a MoM level to predict GDM

Receiver operating characteristic curve analysis showed that the cut-off point of PAPP-A MoM concentration for predicting GDM is 0.995, with a sensitivity of 53.3% and specificity of 51.9%, and the area under the curve was 0.542 (95% CI (0.522–0.563)) (Fig. 1).

However, decreased PAP-A MoM level was related to GDM, there were no significant differences in the prevalence of rupture of membranes (ROM), gestational hypertension, preeclampsia, and pre-term labor between low PAPP-A group and women with normal PAPP-A (Table 4).

**Table 4:** Relationship between PAPP-a MoM level and pregnancy complications

	PAPP-A < 0.4 (n = 322)	PAPP-A ≥ 0.4 (n = 5532)	P value
Gestational diabetes mellitus	69 (21.4)	820 (14.8)	0.001
ROM	46 (14.2)	766 (13.8)	0.826
Gestational hypertension	29 (9.0)	428 (7.7)	0.41
Preeclampsia	14 (4.3)	205 (3.1)	0.555
Pre-term labor	77 (23.9)	1214 (21.9)	0.389

Data are presented as Mean (± SD, range) or n (%) or Median (range), the Pearson chi-square test was utilized to compare the categorical variables among the groups

rupture of membranes (ROM)

## Discussion

It is important to identify pregnant women who are at risk of developing GDM later in pregnancy by using maternal biomarkers in the first trimester and consider preventive intervention for them [19]. We investigated the correlation between plasma protein-A levels (PAPP-A) and GDM, which is a screening marker for aneuploidy that is routinely measured between 11 and 14 weeks of gestation [20]. In this study, the prevalence of GDM among pregnant women was approximately 15% and our results confirmed that maternal PAPP-A MoM concentrations were significantly lower in GDM patients than in normal pregnant women.

However, previous investigations have yielded inconsistent results regarding the relationship between PAPP-A and GDM, possibly due to variations in diagnostic criteria for GDM and sample sizes [21–23].

Our study also found that decreased maternal PAPP-A MoM levels were associated with shorter gestational age at delivery, lower birth weight, and a higher rate of IUGR. This finding is consistent with Bae Hansen et al. study, which demonstrated that pregnant women delivering SGA newborns have significantly lower PAPP-A in the first trimester.

In contrast, we did not find any significant differences in the other pregnancy complications such as ROM, gestational hypertension, preeclampsia, and pre-term labor between the low maternal PAPP-A level group and women with normal PAPP-A concentrations.

Our results are supported by Di Xiao et al.’s retrospective survey on 599 pregnant women with GDM and 986 controls, which revealed that maternal PAPP-a level could be an independent risk factor for GDM development [24]. Furthermore, Wells et al. found that decreased PAPP-A concentration is significantly related to the higher risk of GDM and increased PAPP-A level is associated with a higher risk of LGA [25].

Fruscalzo et al. study on 3263 pregnant women who underwent the first-trimester screening for trisomy between 2005 and 2010 demonstrated women with decreased PAPP-A have a higher risk of subsequent development of diabetes mellitus and short height in offspring [26].

However, a meta-analysis conducted in 2018 revealed that PAPP-A MoM has poor sensitivity to predict GDM. So, it should be combined with other clinical tests to accurately diagnose GDM [14]. Sweeting et al. compared maternal biomarkers, including mean arterial pressure (MAP), uterine artery pulsatility index (UtA PI), PAPP-A, and free-βhCG, between 248 women with GDM and 732 non-GDM. They showed that standard screening factors for aneuploidy and pre-eclampsia could help identify women who subsequently develop GDM, which is cost-effective [27].

A notable advantage of this research is the extensive number of participants included in this study. And we corrected PAPP-A levels for MoM values for maternal characteristics that could be confounding factors.

Our findings suggest that PAPP-A alone has low predictive value for GDM and further investigations are needed to determine the optimal combination of tests to accurately predict GDM.

## Conclusion

Low levels of PAPP-A during early pregnancy increase the risk of glucose intolerance and the development GDM during pregnancy. Additionally, low maternal serum concentrations of PAPP-A are significantly associated with lower birth weight and IUGR.

## Data Availability

The datasets used during the current study available from the corresponding author on reasonable request.
